# Microbial Communities and Organic Matter Composition in Surface and Subsurface Sediments of the Helgoland Mud Area, North Sea

**DOI:** 10.3389/fmicb.2015.01290

**Published:** 2015-11-25

**Authors:** Oluwatobi E. Oni, Frauke Schmidt, Tetsuro Miyatake, Sabine Kasten, Matthias Witt, Kai-Uwe Hinrichs, Michael W. Friedrich

**Affiliations:** ^1^Department of Microbial Ecophysiology, University of BremenBremen, Germany; ^2^MARUM—Center for Marine Environmental Sciences, University of BremenBremen, Germany; ^3^International Max-Planck Research School for Marine MicrobiologyBremen, Germany; ^4^Department of Marine Geochemistry, Alfred Wegener Institute Helmholtz Centre for Polar and Marine ResearchBremerhaven, Germany; ^5^Bruker Daltonik GmbHBremen, Germany

**Keywords:** Helgoland mud area, subsurface sediment, soxhlet extraction, FT-ICR MS, total organic carbon, water-extractable organic matter, Miscellaneous Crenarchaeota Group (MCG), *Chloroflexi*

## Abstract

The role of microorganisms in the cycling of sedimentary organic carbon is a crucial one. To better understand relationships between molecular composition of a potentially bioavailable fraction of organic matter and microbial populations, bacterial and archaeal communities were characterized using pyrosequencing-based 16S rRNA gene analysis in surface (top 30 cm) and subsurface/deeper sediments (30–530 cm) of the Helgoland mud area, North Sea. Fourier Transform Ion Cyclotron Resonance Mass Spectrometry (FT-ICR MS) was used to characterize a potentially bioavailable organic matter fraction (hot-water extractable organic matter, WE-OM). Algal polymer-associated microbial populations such as members of the *Gammaproteobacteria, Bacteroidetes*, and *Verrucomicrobia* were dominant in surface sediments while members of the *Chloroflexi* (*Dehalococcoidales* and candidate order GIF9) and Miscellaneous Crenarchaeota Groups (MCG), both of which are linked to degradation of more recalcitrant, aromatic compounds and detrital proteins, were dominant in subsurface sediments. Microbial populations dominant in subsurface sediments (*Chloroflexi*, members of MCG, and *Thermoplasmata*) showed strong correlations to total organic carbon (TOC) content. Changes of WE-OM with sediment depth reveal molecular transformations from oxygen-rich [high oxygen to carbon (O/C), low hydrogen to carbon (H/C) ratios] aromatic compounds and highly unsaturated compounds toward compounds with lower O/C and higher H/C ratios. The observed molecular changes were most pronounced in organic compounds containing only CHO atoms. Our data thus, highlights classes of sedimentary organic compounds that may serve as microbial energy sources in methanic marine subsurface environments.

## Introduction

Marine sediments cover 70% of the Earth surface. Organic matter is finely dispersed in these sediments in different concentrations depending largely on the size of the organic matter source, water depth, and sedimentation rates (Hedges and Keil, 1995). Apart from organic matter produced in the marine system, e.g., algal and bacterial biomass rich in lipids and nitrogenous compounds, marine sediments also receive inputs of terrestrial organic matter, which is mainly derived from plant materials rich in cellulose and lignin (De Leeuw and Largeau, 1993). Regardless of sources, extensive recycling of organic matter occurs in the water column (Hedges and Keil, 1995) and only about 1% of the organic carbon export reaches the seafloor on a global scale (Hedges and Keil, 1995). This detrital organic matter serves as a main energy source for microorganisms living in marine sediments (Jørgensen and Boetius, 2007).

In surface sediments, easily degradable organic matter is preferentially utilized by microorganisms (Cowie and Hedges, 1994; Wakeham et al., 1997), whereas less reactive organic matter accumulates and is buried in deeper sediments (Zonneveld et al., 2010). Consequently, microorganisms inhabiting deeper sediments have to meet their metabolic demands by relying on more recalcitrant organic matter, whose degradation requires longer time scales (Middelburg, 1989; Biddle et al., 2006). There are very few studies (e.g., Xie et al., 2013; Vigneron et al., 2014) on the nature of organic matter mineralized by microorganisms in marine subsurface sediments. However, the consistence of microorganisms dominating subsurface sediments across many environments may be due to special adaptations for utilization of less reactive organic matter (Biddle et al., 2006; Inagaki et al., 2006). Dominant Bacterial phyla are usually *Chloroflexi* and candidate division JS1 (Inagaki et al., 2006; Webster et al., 2007; Hamdan et al., 2011), while dominant Archaea are mostly members of the Miscellaneous Crenarchaeota Group (MCG) and Marine Benthic Group B (MBGB), otherwise referred to as Deep Sea Archaeal Group (DSAG; Biddle et al., 2006; Inagaki et al., 2006; Teske and Sørensen, 2008; Kubo et al., 2012). How these important groups of microorganisms thrive, and what carbon sources they assimilate is largely unknown.

Knowledge of the molecular composition of sedimentary organic matter is important to predict the contributions of different organic matter sources to the pool of total organic carbon (TOC; Meyers and Ishiwatari, 1993), each pool's relevance for shaping the functional diversity of microbial communities (Hunting et al., 2013) and associated energy limitations originating from substrate composition (Lever et al., 2015). However, it is a major challenge to molecularly characterize organic matter in sediments due to analytical limitations (Nebbioso and Piccolo, 2012). In the last decade, Fourier Transform Ion-Cyclotron Resonance Mass Spectrometry (FT-ICR MS) has successfully provided insights into the molecular composition of dissolved organic matter (DOM) in diverse environments (Kim et al., 2004; Koch et al., 2005; Dittmar and Koch, 2006; Hertkorn et al., 2006; Tremblay et al., 2007; Reemtsma et al., 2008; Schmidt et al., 2009, 2014; Bhatia et al., 2010; D'Andrilli et al., 2010; Lechtenfeld et al., 2013; Roth et al., 2013; Kellerman et al., 2014; Seidel et al., 2014; Dubinenkov et al., 2015) due to its capacity to resolve thousands of individual components of complex organic matter based on accurate mass measurement. We applied FT-ICR MS to the water-extractable organic matter (WE-OM) fraction, which consists of free and adsorbed pore-water DOM as well as DOM that can be leached from particulate organic matter (Schmidt et al., 2014). Thus, WE-OM is representative of both pore-water DOM and its potential particulate precursor pool. This pool of organic matter may also provide utilizable carbon and nitrogen for microorganisms living in sediments and soils (Strosser, 2010; Guigue et al., 2015). However, the ubiquity, distribution, and potential relevance, as a substrate source, of individual groups of DOM molecules for microbes in marine sediments are not known.

The Helgoland mud area (German Bight of the North Sea) is one of the depocenters of fine-grained mud in the open North Sea. In periods before 1250 A.D., this area has experienced higher sedimentation rates (up to 12-fold higher) and deposition of organic matter than now-a-days (Hebbeln et al., 2003). With this work, we aim at a molecular characterization of WE-OM and prokaryotic communities in sediments from the Helgoland mud area and discuss potential links between the molecular composition of organic matter and diversity of microbial populations in marine sediments.

## Methods

### Site and sampling description

Samples from surface sediments (up to 10 cm) and deeper sediments (up to 530 cm) from the Helgoland mud area (54° 5.00′N 7° 58′E) were collected in 2012, 2013, and 2014 during cruises with the research vessels HEINCKE and UTHÖRN. Sampling sites, coordinates, and methods are described in detail by Oni et al. (2015). Microbial community analysis was performed on samples reported in the aforementioned study. For sediment cores collected in 2012 (core UT2012, surface sediments and core HE376-007, deeper sediments), TOC, total nitrogen (TN), stable carbon, and nitrogen isotope analysis was performed with samples from 0 to 5, 5 to 10 cm, and each 25 cm sections of the 500 cm sediment core described in Oni et al. (2015). The same parameters were measured on sediment cores collected in 2013 (core HE406-8-003, deeper sediments). From sediment core HE421-004, only 4–6 cm (surface sediments) was sampled while sediment core HE406-8 was sampled in 25 cm sections at 100 cm intervals [i.e., 30–55 cm (close to the sulfate-methane transition depth, SMT (75 cm, Oni et al., 2015), termed “SMT area” hereafter), 130–155, 230–55, 330–355, and 430–455 cm (methanic zone)]. Samples from cores HE421-004 and HE406-8 were used for studying the molecular composition of organic matter by aqueous soxhlet extraction and subsequent FT-ICR MS analysis of extracts.

### Organic matter analysis

#### Total organic carbon, total nitrogen, and stable carbon and nitrogen isotopes

To quantify the contents of TOC, TN, and their respective stable isotopes, approximately 3 g of wet sediment from each section was decalcified by treatment with 10% HCl. Afterwards, samples were washed with ultrapure water and freeze-dried followed by grinding in a mortar. 10–30 mg of each sample was weighed into tin capsules and analyzed on a Thermo Scientific Flash 2000 elemental analyzer connected to a Thermo Delta V Plus IRMS. All values are mean values of duplicate measurements. Stable isotopic compositions (δ^13^C and δ^15^N) are reported in ‰ relative to the Vienna Pee Dee Belemnite (V-PDB) standard and atmospheric N respectively. High-resolution TOC contents were determined using a Carbon-Sulfur Determinator (ELTRA CS 2000). About 50 mg of dried and ground sediment were weighted into ceramic crucibles. Two to three drops of ethanol were added to avoid strong bubbling. Subsequently, the sediment was decalcified with 12.5% HCl p.a. and dried on a heating plate at 250°C. After about 2 h, the dry sediment was covered by a mixture of steel and tungsten splinters to ensure a homogenous burning of the sample. The analytical precision was better than 1%.

#### Soxhlet extraction

A detailed description of extraction procedures and post-extraction steps has been provided in Schmidt et al. (2014). In brief, about 25 g of wet sediment was weighed into pre-combusted glass fiber thimbles (30 × 100 mm, Whatman). Prior to use, thimbles were extracted in ultrapure water for 48 h to remove potential contaminants. A procedural blank containing thimble and deionized water was run to check for contaminations. The thimbles were placed in the soxhlet extraction unit and WE-OM was extracted from the sediment samples with 200 ml of distilled, de-ionized water for 24 h. Soxhlet extracts were filtered first with 0.7 μm (GF/F, Whatmann) and then 0.2 μm (cellulose acetate, Sartorius) microbiologically sterile filters before storing extracts at 4°C until further use.

#### DOM extraction

Soxhlet extracts were acidified to pH 2 with HCl (suprapur, Merck) before concentrating the DOM by solid phase extraction (SPE) using Bond Elut-PPL cartridges (500 mg, 3 ml syringe; Agilent Technologies, Germany) as described by Dittmar et al. (2008). As the extracts were adsorbed to the cartridges, salts were removed by rinsing the cartridges with 6 ml ultrapure water (pH 2). Extracts were eluted with 1 ml of methanol (LiChrosolv, Merck) and stored at −20°C in the dark until FT-ICR MS analyses.

#### Dissolved organic carbon and total dissolved nitrogen

DOC and total dissolved nitrogen (TDN) concentrations were analyzed in Soxhlet extracts and SPE extracts. First, methanol was removed from aliquots of SPE extracts under a stream of nitrogen and afterwards DOM was re-dissolved in 6 ml ultrapure water. Measurements were performed by high-temperature catalytic oxidation (at 680°C) using a Shimadzu TOC/TN analyzer equipped with infrared and chemiluminescence detector (oxygen flow: 0.6 l min^−1^). Prior to direct injection onto the catalyst samples were acidified with 0.12 ml HCl (2 M) in the autosampler and purged with oxygen to remove inorganic carbon. Final DOC and TDN concentrations were average values of triplicate measurements.

#### FT-ICR MS

DOM extracts were analyzed on a Bruker SolariX XR FT-ICR mass spectrometer (Bruker Daltonik GmbH, Bremen, Germany) equipped with a 12 T refrigerated actively shielded superconducting magnet (Bruker Biospin, Wissembourg, France), a dual ionization source (ESI and MALDI, Apollo II electrospray source, Bruker Daltonik GmbH, Bremen, Germany) and a dynamically harmonized analyzer cell (ParaCell™, Bruker Daltonik GmbH, Bremen, Germany). Prior to measurement, the extracts were diluted with methanol:water (1:1, v/v) mixture to same SPE concentrations for all samples (750 nmol DOC/mL). Samples were ionized using electrospray ionization in negative ionization mode at an infusion flow rate of 5 μl min^−1^. Ion accumulation time was set to 0.05 s and 200 scans were added to one mass spectrum. Mass spectra were acquired with 4 MW data points resulting in a resolving power of 480,000 at m/z 400. Mass spectra were calibrated externally with arginine clusters and recalibrated internally with compounds that were repeatedly identified in marine pore-water DOM samples (cf. Schmidt et al., 2014). The root mean square error of the internal calibration was below 0.095 ppm resulting in very reliable molecular formula assignment. Molecular formulas were calculated under consideration of the following elements ^1^H_0−90_, ^12^C_0−60_, ^13^C_0−1_, ^16^O_0−35_, ^14^N_0−4_, ^32^S_0−2_, ^34^S_0−1_, ^31^P_0−2_ in a m/z range of 180–600 using a custom-developed software written in C^++^.

Formulas were restricted to integer double bond equivalent (DBE) values and a molecular element ratio of O/C ≤ 1.2. A mass tolerance of ±0.5 ppm was considered as a valid formula. Multiple formulas were filtered with the homologous series/building block approach and isotope check (Koch et al., 2007). Molecular formulae containing ^13^C or ^34^S were excluded from the final dataset which was limited to peaks with S/N > 7 corresponding to a relative peak intensity of 0.4%. Relative peak intensities were calculated from the total peak intensity (ΣInt_allPeaks_) in the spectra after following equation:

(1) $$\text{Rel}.\text{ Intensity}=({\text{Int}}_{\text{Peak}}\text{/}\Sigma {\text{Int}}_{\text{allPeaks}}{}^{*}\text{1000})$$

In order to reduce the complexity of data characteristically obtained from FT-ICR MS analyses, molecular formulae were first grouped into categories based on their elemental composition: (1) molecular formulae containing C, H, and O atoms, (2) molecular formulae consisting of C, H, O, and one or two N atoms (CHO-N_1−2_), (3) molecular formulae consisting of C, H, O, and three or four N atoms (CHO-N_3−4_), (4) molecular formulae containing N and P (CHNOP), (5) molecular formulae containing S (CHOS), (6) molecular formulae containing N and S (CHNOS), (7) molecular formulae containing P and S (CHOPS) as well as (8) those containing P only (CHOP). In addition, molecular formulae in the different categories were divided into five groups based on modified aromaticity index (AI_mod,_ Koch and Dittmar, 2006), H/C and O/C ratios (e.g., Šantl-Temkiv et al., 2013; Seidel et al., 2014), hereafter referred to as groups 1–5: (group 1) polycyclic aromates, (PCAs, AI_mod_ ≥ 0.67), (group 2) highly aromatic compounds, including polyphenols and PCA compounds with aliphatic chains (0.67 > AI_mod_ > 0.50), (group 3) highly unsaturated compounds (including humic compounds and carboxyl-rich alicyclic molecules (CRAM; Hertkorn et al., 2006; AI_mod_ ≤ 0.5 and H/C < 1.5), (group 4) unsaturated aliphatic compounds (2.0 > H/C ≥ 1.5), (group 5) saturated aliphatic compounds (may include carbohydrate-like compounds, saturated fatty and sulfonic acids; H/C ≥ 2.0). Raw data sheets used for molecular assignments are provided as Supplementary Material (Data Sheet S4). ESI negative FT-ICR mass spectra covering all mass ranges in WE-OM and detailed mass spectra on nominal mass 385Da (as an example) are also provided in Figures S1, S2, respectively.

### Microbial community analyses

#### Pyrosequencing and sequence analyses

DNA samples extracted as described in Oni et al. (2015), from depths 0 to 5 and 5 to 10 (surface sediments), 30 to 55 (SMT area), 180 to 205, 230 to 255, 305 to 330, 355 to 380 and 480–505 cm (methanic zone), were selected for 454 FLX pyrosequencing at Molecular and Research Testing Laboratory (Lubbock, Texas, USA). Same primer pairs for bacterial and archaeal 16S rRNA gene amplification as reported in Oni et al. (2015) were used. Downstream processing of sequence raw data files (SFF files) were done as reported earlier (Oni et al., 2015). Rarefactions curves (observed species based on 97% OTU cut-off) and microbial diversity and microbial and richness indices (Shannon and Chao 1, Hughes et al., 2001; Spellerberg and Fedor, 2003) were calculated for each sample analyzed using QIIME version 1.7.0. Species diversity and richness indices along the depth profile for bacteria and archaea were calculated after normalizing the number of sequences to those of the samples with lowest sequence reads. Weighted Paired Group Method of Averaging (WPGMA) cluster diagrams were generated for bacterial and archaeal OTUs.

#### Statistical analyses

To investigate the strength of relationships between TOC and TN or between TOC and microbial populations with depth, spearman correlations were calculated using PAleotontological STatistics software version 2.17c (PAST, Hammer et al., 2001).

## Results

### Organic matter analyses

#### Total organic carbon, total nitrogen, and stable carbon and nitrogen isotopes

**Figure 1:**
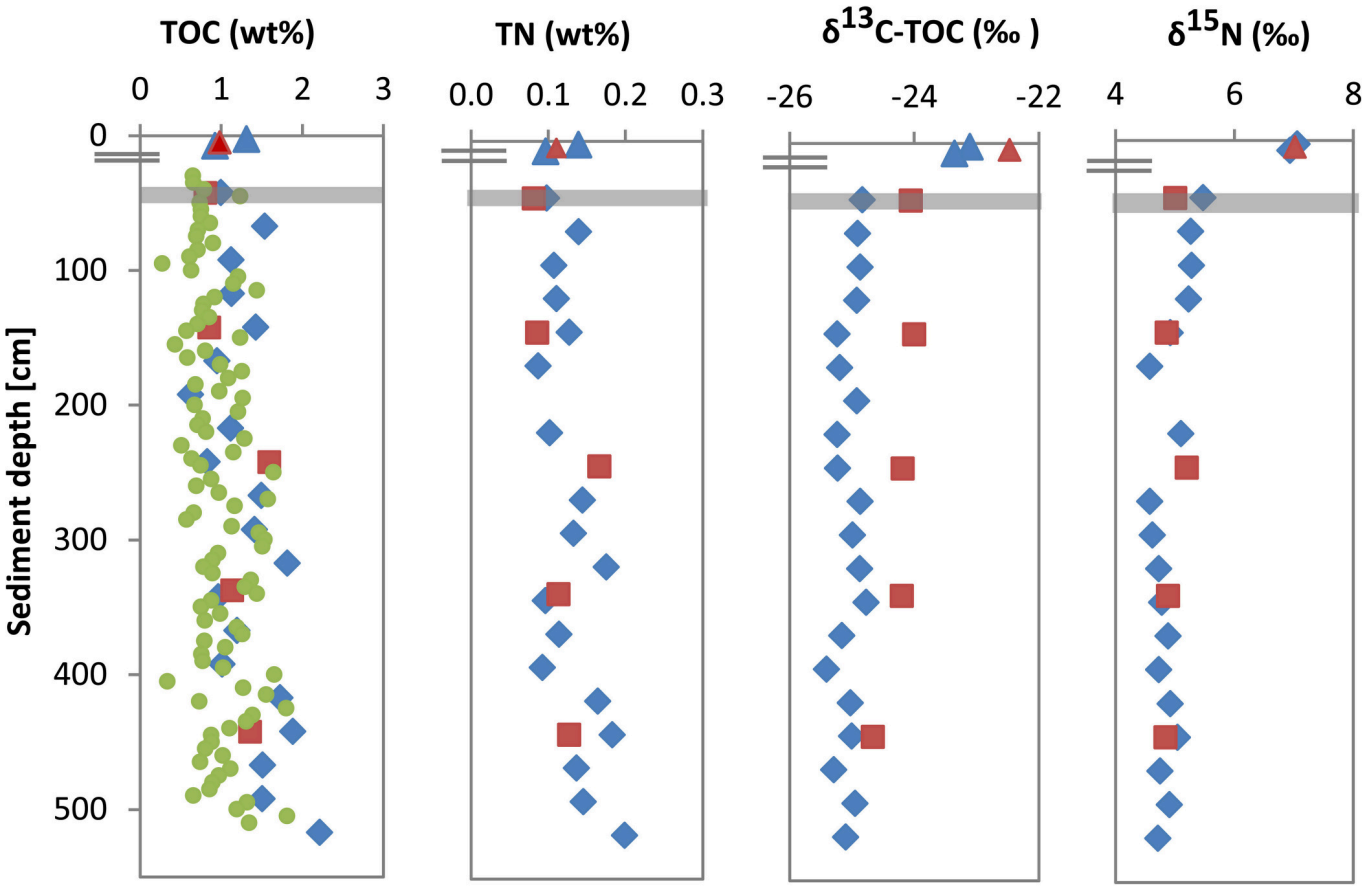
**Depth profiles of TOC, TN, stable carbon, and stable nitrogen in surface and subsurface sediments of the Helgoland mud area**. Surface sediment; core UT2012 (blue triangle), surface sediment; core HE421-004 (brown triangle), deep sediment; core HE376-007-5 (blue diamond), deep sediment; core HE376-007-2 (high-resolution TOC, green dots), deep sediment; core HE406-008 (brown square). Gray bar represents SMT.

In cores UT-2012 (surface sediments) and HE376-007-5 (deeper sediments), mean TOC and TN values showed high variations with depth (Figure 1). TOC and TN contents ranged between 0.6–2.2 and 0.09–0.2 wt % respectively, with the highest values of both parameters measured at depths below 300 cm. Depth-wise TOC and TN variations strongly co-varied (Figure 1; ρ = 0.962, *p* = 1.48E–11, *n* = 22). δ^13^C-TOC and δ^15^N values ranged between −23.1 to −23.4‰ and 6.9 to 7.1‰ in the surface sediment. In deeper sediments, δ^13^C-TOC and δ^15^N values showed variations between −24.9 to −25.4 and 4.6 to 5.5‰, respectively (Figure 1). In cores HE421-004 and HE406-008, TOC, TN, δ^13^C-TOC, and δ^15^N distributions were similar to those of cores UT-2012 and HE376-007-5. In core HE421-004, TOC, and TN in the surface sediment (4–6 cm) were 0.98 and 0.11 wt %, respectively. In deeper sediments, TOC varied between 0.81 and 1.6 wt % and TN ranged from 0.08 to 0.17 wt %. Both parameters showed the same trend with depth (ρ = 1.000, *p* < 0.001, *n* = 6) with the highest values observed in sediments sampled below 230 cm (Figure 1). Furthermore, both δ^13^C-TOC and δ^15^N gradually decreased with depth (Figure 1).

#### Water-extractable organic matter analysis

**Figure 2:**
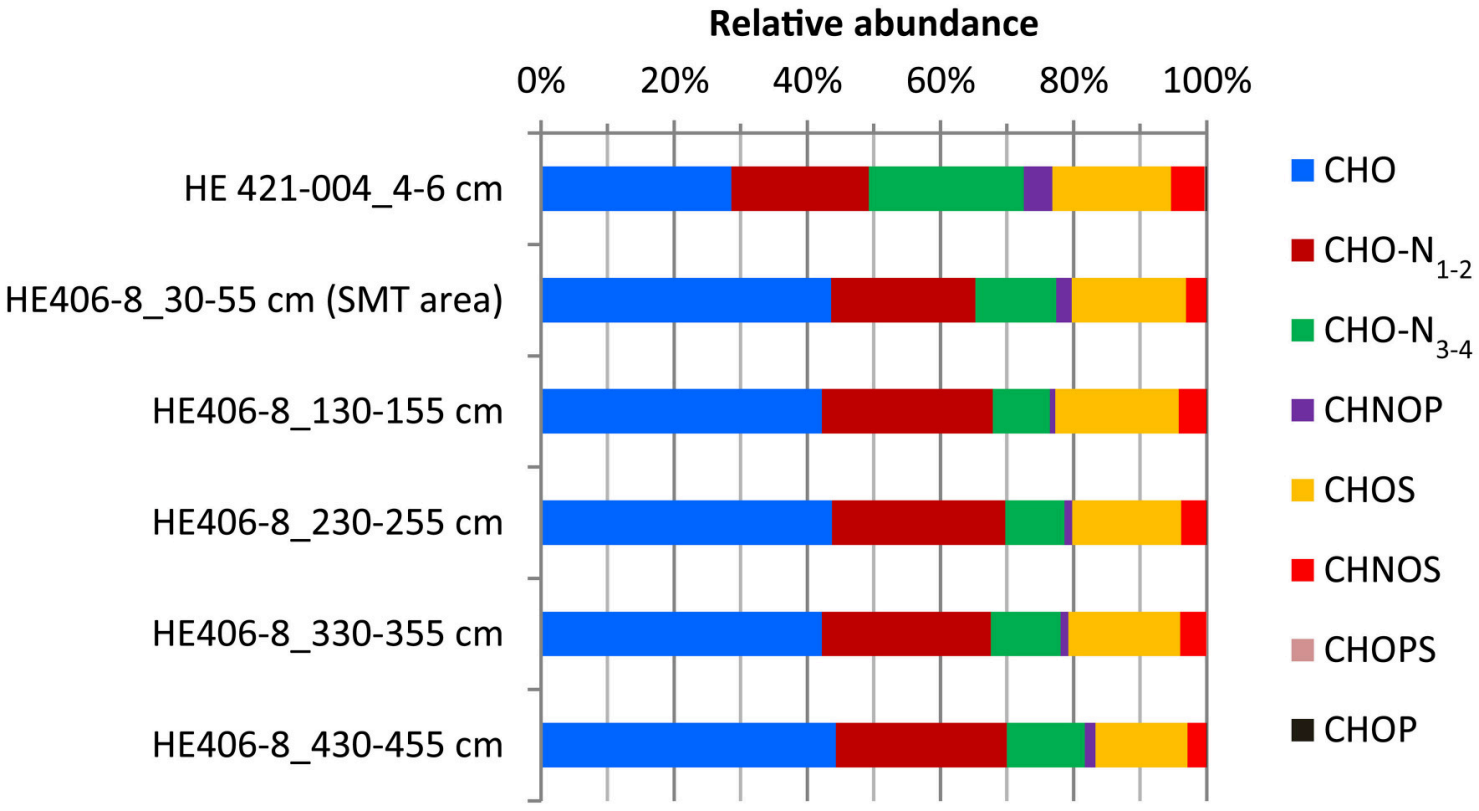
**Depth-wise relative abundance distribution of intensities of molecular formulae groups classified based solely on heteroatoms (N, S, and P) contents**. Surface sediment samples (4–6 cm) are obtained from core HE421-004. Deeper sediments samples (30–455 cm) are obtained from core HE 406-008.

WE-OM fraction in the surface and deeper sediments ranged between 1.2 and 2.4% (Table 1) with the highest portion found for the sample from 130 to 155 cm and the lowest portion for the sample from 230 to 255 cm. FT-ICR MS analysis resolved thousands of molecular formulae per sample (Table 2). The sample from the surface sediment (4–6 cm) contained a lower number of formulae compared to samples from deeper sediments (30–455 cm). In deeper sediments, numbers of molecular formulae were higher in the samples from the methanic zone (below 130 cm) compared to the sulfate methane transition zone (SMT area; 30–55 cm). Intensity weighted averages of molecular masses (m/z_wa_) were higher in deeper sediments than in surface sediments. Weighted average Double Bond Equivalent (DBE_wa_) values, which denote the sum of rings and double bonds in the molecular compounds, as well as O/C_wa_ and C/N_wa_ ratios, were generally lower in surface sediments. Conversely, H/C_wa_ ratio was higher in the surface sediment compared to the deeper sediments. With respect to relative intensities of peaks, total signal intensities of CHO and N-bearing compounds were highest in all samples. CHO and CHO-N_1−2_ compounds were more enriched in deeper sediments whereas CHO-N_3−4_ and CHNOP compound groups were most abundant in the surface sediments (Figure 2). Relative signal intensities of CHOS compounds showed no clear trend from surface sediments down to deeper sediments (Figure 2).

**Table 1:** **Concentrations of dissolved organic carbon (DOC), total dissolved nitrogen (TDN), their ratios in WE-OM, and proportion of water extractable organic carbon (WE-OC) in TOC**.

**Sample**	**DOC (μM)**	**TDN (μM)**	**DOC/TDN**	**WE-OC (mg C/g sed.)**	**TOC (%)**	**TOC (mg/g sed.)**	**WE-OC (%TOC)**
HE421-004_4–6 cm	1103.94	134.82	8.19	0.20	0.98	9.79	2.05
HE406-8_30–55 cm	827.34	64.90	12.75	0.14	0.81	8.06	1.72
HE406-8_130–155 cm	913.74	88.62	10.31	0.20	0.86	8.55	2.38
HE406-8_230–255 cm	1078.14	113.82	9.47	0.20	1.60	15.95	1.23
HE406-8_330–355 cm	976.74	103.32	9.45	0.19	1.14	11.36	1.65
HE406-8_430–455 cm	1468.47	154.47	9.51	0.25	1.36	13.55	1.84

**Table 2:** **Number of molecular formulae, weighted averages (wa) of DBE, molar ratios of oxygen, hydrogen, carbon, nitrogen atoms, and charge to mass ratios of water-extractable organic matter as obtained from FT-ICR MS analysis**.

**Sample**	**Number of formulae**	**DBE_wa_**	**H/C_wa_**	**O/C_wa_**	**C/N_wa_ ratio**	**m/z_wa_**
HE421-004_4–6 cm	4858	7.22	1.35	0.47	12.83	367.02
HE406-8_30–55 cm	5805	8.88	1.17	0.52	21.92	389.44
HE406-8_130–155 cm	6348	8.42	1.20	0.54	24.46	391.95
HE406-8_230–255 cm	6899	8.53	1.21	0.51	24.55	392.20
HE406-8_330–355 cm	6780	8.43	1.21	0.52	22.47	391.04
HE406-8_430–455 cm	6936	8.70	1.20	0.50	22.01	391.01

**Figure 3:**
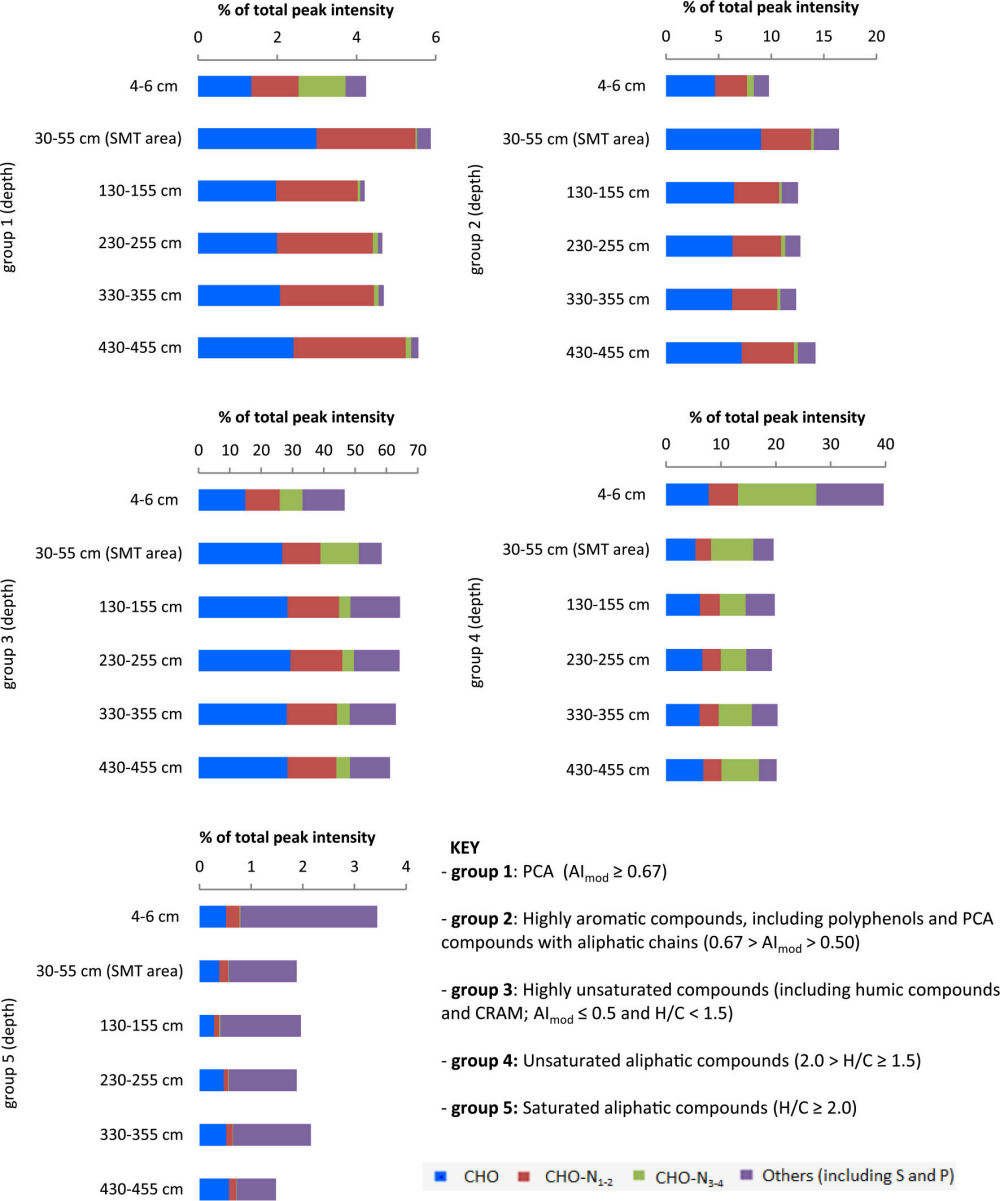
**Depth-wise relative abundance distribution of intensities of compound groups classified based on modified aromaticity index (AI_mod_), H/C and O/C ratios**. At each depth, compound groups are further divided based on heteroatoms (N, S, and P). Surface sediment samples (4–6 cm) are obtained from core HE421-004. Deeper sediments samples (30–455 cm) are obtained from core HE 406-008.

**Figure 4:**
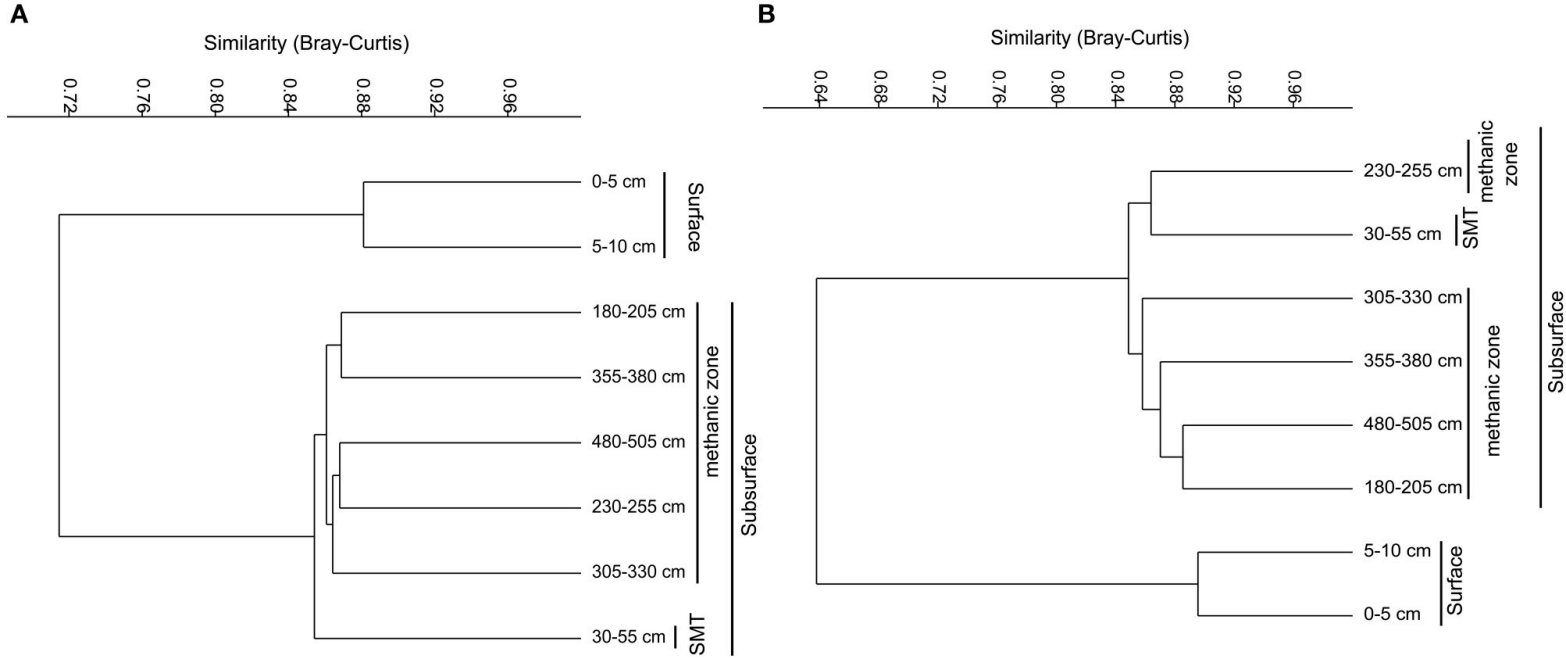
**Weighted Paired Group Method of Averaging (WPGMA) cluster diagrams of bacterial (A) and archaeal (B) OTUs (97% cut off) obtained from pyrosequencing-based 16SrRNA gene sequencing**.

PCAs (group 1) showed the highest relative abundance in the sample from the SMT area (4.1%), followed by sample from the surface sediment (3.8%). Their abundance decreased at 130–355 cm and below, where it ranged between 2.5 and 2.7%. In the deepest sample from 430 to 455 cm PCA compounds showed a slight increase to 3.4% (Figure 3). Highly aromatic compounds (group 2) were comparatively more abundant in all samples and showed similar trends in deeper sediments as group 1 (Figure 3). The decrease in the percentage relative intensities of PCA and highly aromatic compounds below the SMT area appeared to be most pronounced in the CHO compounds (Figures 3, 4B). Highly unsaturated compounds (group 3) were the most abundant molecular formulae group in all samples (Figure 3). In the surface sediment they constitute 47% of all peak intensities while their relative abundance increased in deeper sediments, from 58% in the SMT area to 61–64% in the methanic zone. Unsaturated aliphatic compounds (group 4) were highest in the surface sediment (40%) whereas their relative intensities decreased in the deeper sediment to approximately half (19.3–20.3%) of their total intensities in the surface sediment. The relative abundances of CHO-N_3−4_ in surface sediments, were most abundant in group 4 and group 1 (Figure 3). Finally, saturated aliphatic compounds (group 5) were most abundant in the surface sediment (3.4%) in relation to samples from deeper sediments (1.4–2.2%). CHO-N_3−4_ formulae made up a small portion (~0.3–1%) of the compounds in group 5 (Figure 3).

### Microbial community structure and composition

**Figure 5:**
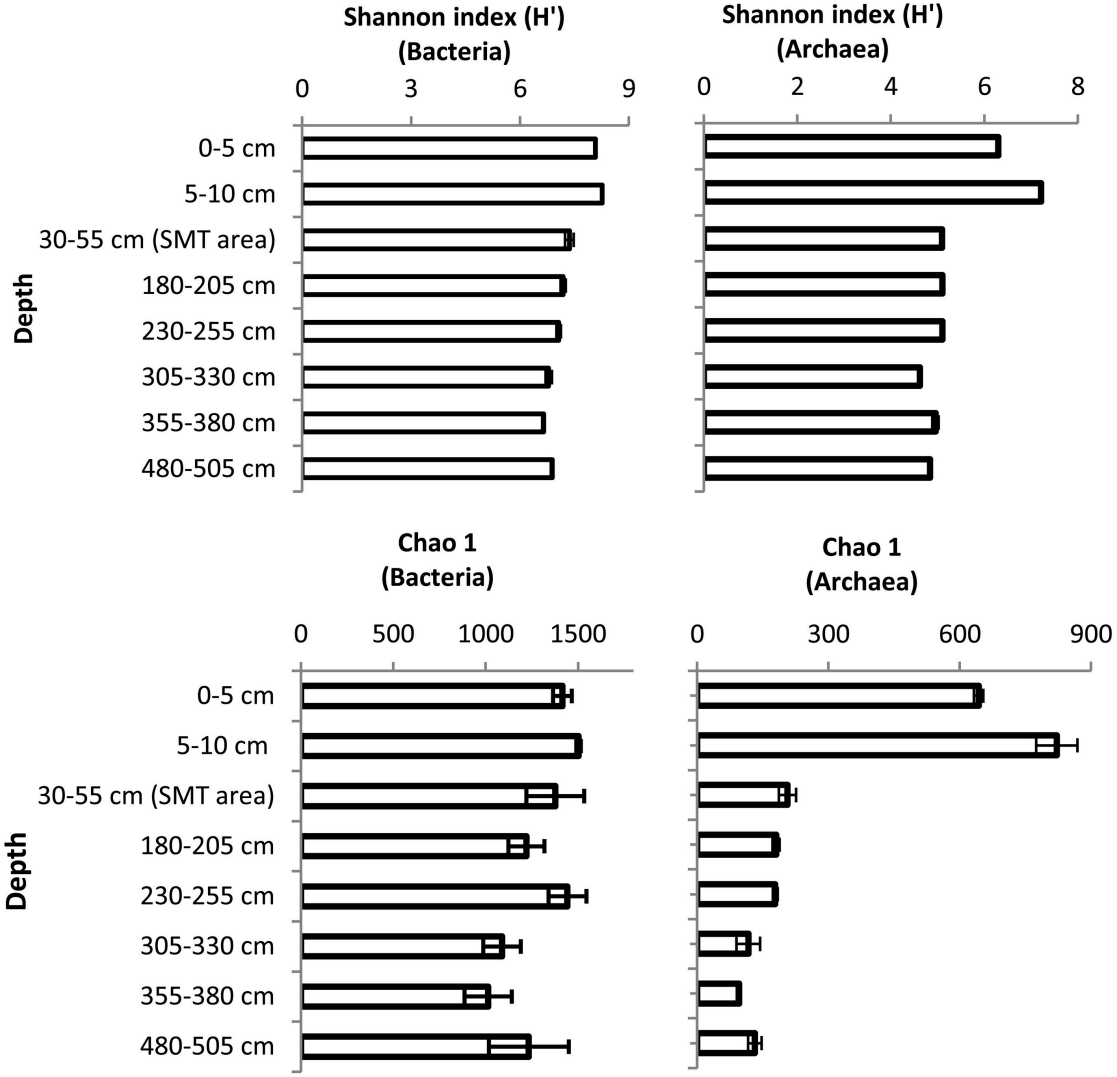
**Shannon and Chao1 diversity indices of Bacteria and Archaea in surface and deeper sediments of the Helgoland mud area**. Error bar are standard deviations for results of calculations of diversity indices over three iterations.

**Figure 6:**
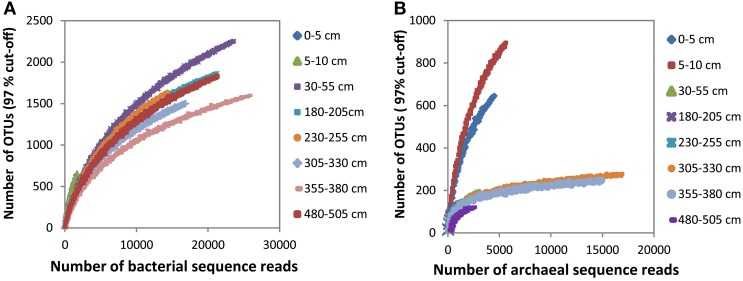
**Rarefaction curves of bacterial (A) and archaeal (B) OTUs (97% cut-off) detected at the depths sampled in the surface and deeper sediments**.

The bacterial and archaeal community structure clearly differed between the surface and deeper sediments as displayed in Figures 4A,B. Specifically, deeper sediments showed a separation between bacterial populations in the SMT area and the methanic zone (Figure 4A). However, there was no separation of archaea between the SMT area and the methanic zone (Figure 4B). Bacterial and archaeal diversities (Shannon index) were higher in surface compared to deeper sediments (Figure 5). Overall, no clear differences in bacterial species richness were observed between surface and deeper sediments (Figure 5). However, archaea species richness was approximately 4–9 times higher in surface than in deeper sediments (Figure 5). Estimates of the number of bacterial and archaeal OTUs detected (based on 97% sequence similarity cut-off) are shown in rarefaction curves (Figures 6A,B).

**Figure 7:**
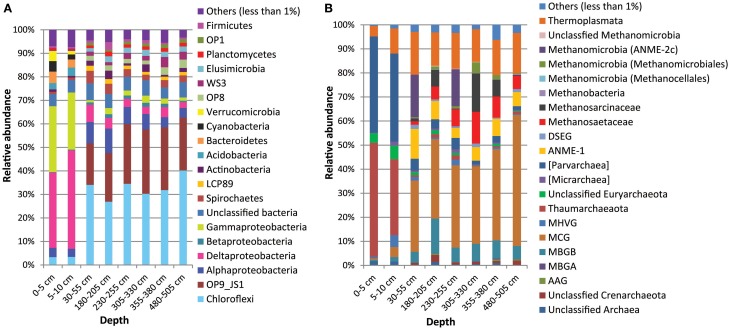
**Composition of Bacterial (A) and Archaeal (B) communities in the Helgoland mud area based on pyrosequencing analyses of 16S rRNA gene data from core HE376-007-5**.

In comparison to deeper sediments, bacterial communities in the surface sediments were dominated by *Deltaproteobacteria* (33-34% in surface *vs*. 2.5–7% in deeper sediments) and *Gammaproteobacteria* (25–29% in surface *vs*. 1–3% in deeper sediments). Deeper sediments were dominated by *Chloroflexi* (27-40 *vs*. 3% in surface sediments) as well as candidate division OP9/JS1 (18–27%; not detected in surface sediments). Bacterial populations belonging to *Acidobacteria* (3–4%), *Verrucomicrobia* (1.5–4.4%), Cyanobacteria (2–4.5%), and *Bacteroidetes* (3.5–5%) were more dominant in the surface sediments (Figure 7A). In contrast, *Spirochaetes* (2.3–5.3%), salt marsh clone LCP89 (1.22–2.19%), *Betaproteobacteria* (0.6–1.7%), *Planctomycetes* (1.2–2.0%), *Elusimicrobia* (1.7–2.6%), candidate divisions OP1 (0.71–1.6%), OP8 (1.7–3.6%), and WS3 (1.3–4.4%) were more abundant in all samples from deeper sediments compared to surface sediments (Figure 7A). Other bacterial populations such as *Actinobacteria* and *Firmicutes* were detected both in surface and deeper sediments with more or less similar relative abundances (Figure 7A).

Archaeal populations in surface sediments were largely dominated by *Thaumarchaeota* (31–47% in surface *vs*. 0.7–1.9% in deeper sediments) and Parvarchaea (36–40% in surface vs. 1.5–5% in deeper sediments) while deeper sediments were dominated by MCG (30–56% in deeper *vs*. 0.6–4% in surface sediments) (Figure 7B). Anaerobic methanotrophic archaea (ANME 1 and ANME-2c) were detected in the deeper sediments with their combined relative abundances highest at 30–55 cm (SMT area). Methanogens belonging to the *Methanosaetaceae, Methanosarcinaceae* and *Methanomicrobiales* were more abundant in the deeper sediments, in particular in the methanic zone (305–330 and 355–380 cm) (Figure 7B). Conspicuously, more abundant in samples from deeper sediments were the MBGB (4.7–15% in deeper *vs*. 0.5–1.9% in surface sediments) and *Thermoplasmata* (13–17% in deeper *vs*. 4–10% in surface sediments).

Up-to-family-level relative abundance information on bacterial and archaeal populations at each sampled depth are given in Data Sheets S1, S2, respectively.

### Organic matter-linked microbial populations in deeper sediments

**Figure 8:**
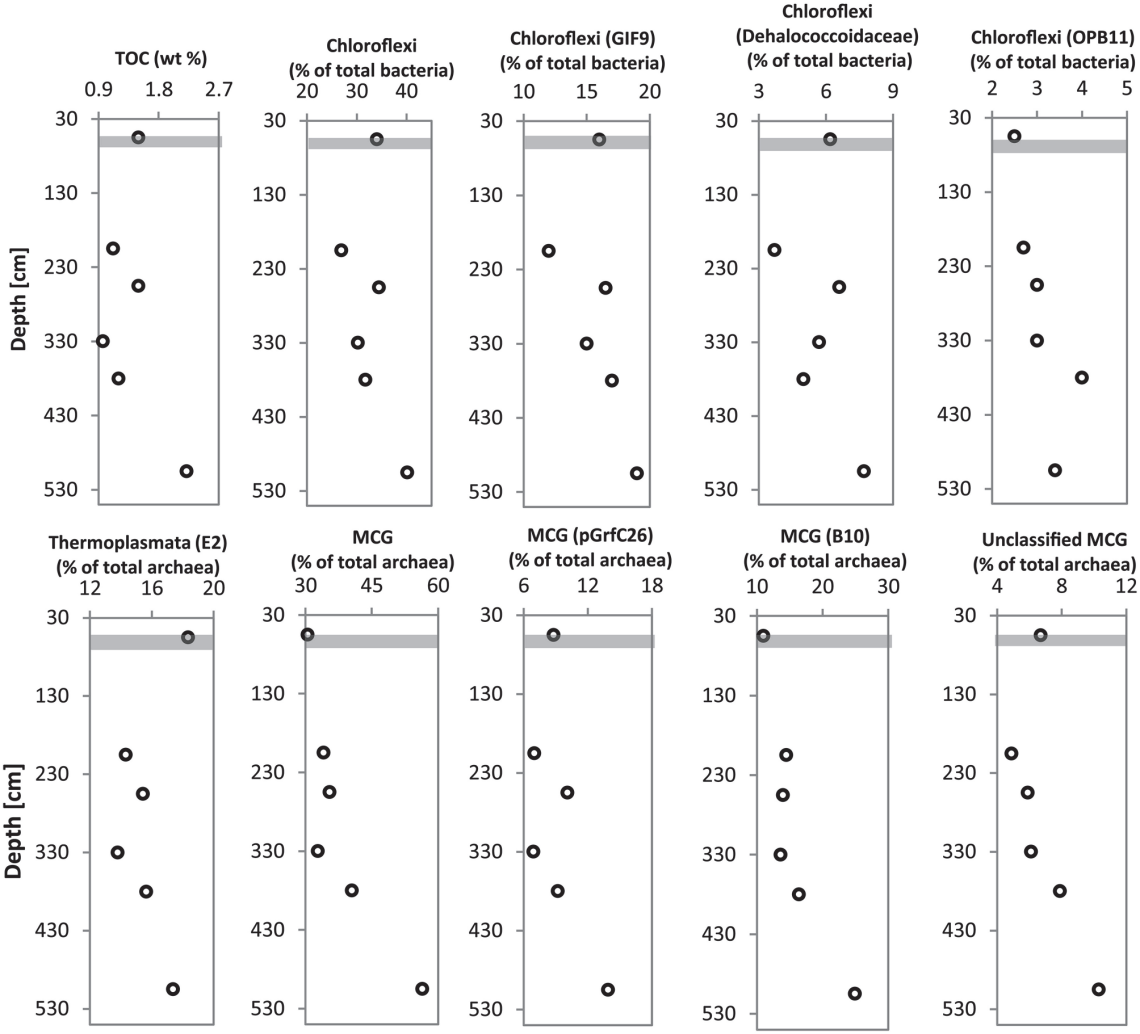
**Depth profiles of dominant Bacteria (*Chloroflexi*) and Archaea (*Thermoplasmata* and MCG) in relation to TOC content at sediment depths from which DNA was extracted for pyrosequencing analysis of 16S rRNA genes**. Gray bar represents SMT.

Multiple sediment samples retrieved from the gravity core (HE 376-007-5) allowed the possibility to match the depth-wise distribution of bacterial and archaeal populations detected in deeper sediments to TOC content at depths from which samples were chosen for microbial molecular analysis. Microbial populations belonging to *Chloroflexi* (ρ = 0.928, *p* = 0.01; mainly *Dehalococcoidales*, candidate order GIF9), *Thermoplasmata* (ρ = 0.812, *p* = 0.07), and a candidate order of the MCG (pGrfC26; ρ = 0.899, *p* = 0.03) showed strong correlations to TOC (Figure 8, Data Sheet S3).

## Discussion

We characterized the molecular composition of the WE-OM pool of bulk organic matter in the surface and deeper sediments. In addition, prokaryotic community composition of the Helgoland mud area was studied. Our findings, as discussed below, reveal important differences in the molecular composition of WE-OM and organic matter bioavailability, which may play a role in determining microbial populations dominating in surface and deeper sediments.

### Sources and bioavailability of organic matter in surface sediments

**Figure 9:**
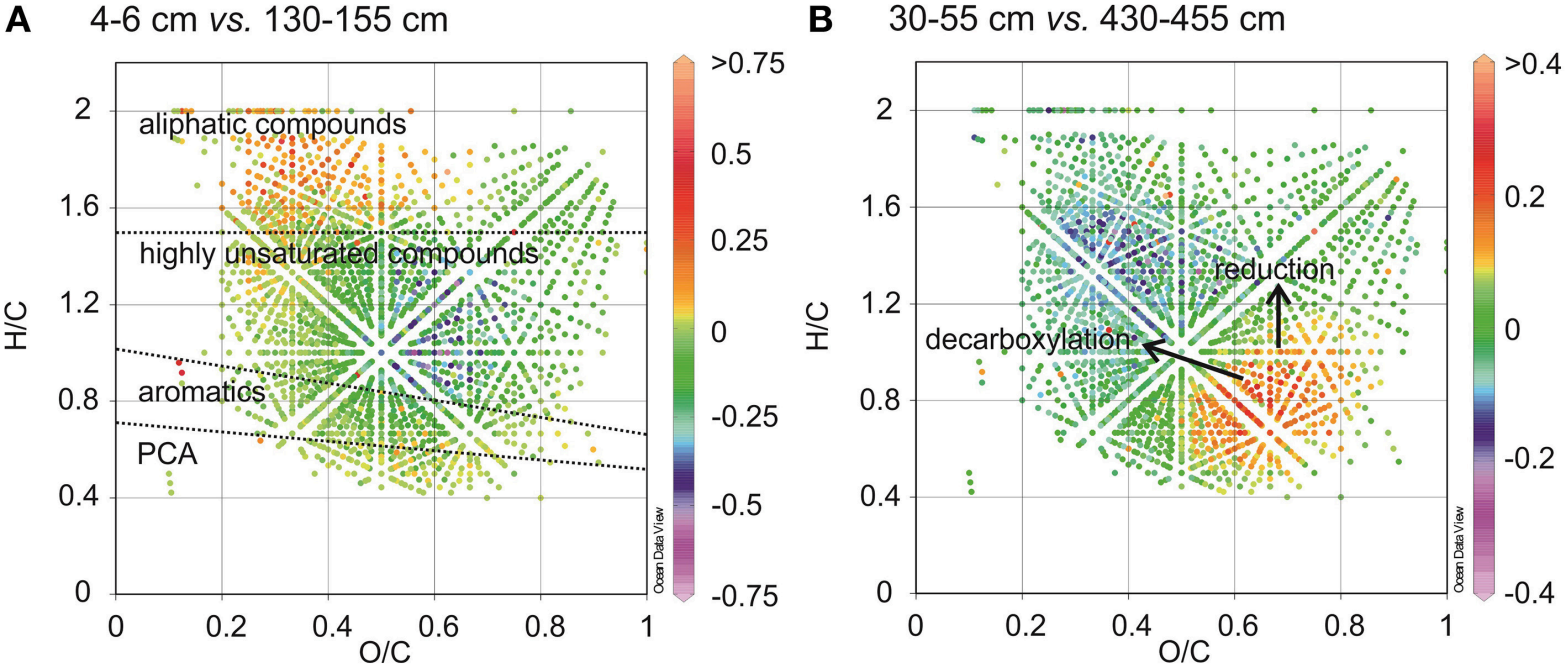
**Differential van Krevelen diagrams compare the relative formula intensities of CHO compounds between two samples showing molecular variations between surface and subsurface sediments (A), and between two subsurface sediments (B)**. Higher formula intensities in each of the shallower samples relative to the deeper samples is indicated by positive values (orange to red color) whereas negative values (blue to purple color) indicate higher formula intensities in the deeper samples relative to the shallow ones.

The relative ^13^C enrichment of organic matter (δ^13^C of TOC is −23.1 to −23.4‰) in surface sediments is indicative of higher contributions of marine derived organic matter such as algal materials (Dauwe and Middelburg, 1998; Holtvoeth, 2004; Sangiorgi et al., 2005). Algal organic matter consists of a higher portion of aliphatic and N-rich molecules (Sun et al., 1997). It has previously been shown that near-surface pore-water DOM from open marine sites with a predominance of algal material, contains more molecular formulae with N and elevated H/C ratios (Schmidt et al., 2009). In line with this were the low C/N_wa_ and high H/C_wa_ ratios (Table 2) and higher abundances of saturated and unsaturated aliphatic compounds (groups 4 and 5, Figure 3) in WE-OM from the surface sediment. In the van Krevelen diagram (Figure 9A), the difference in the CHO formulae between surface and deeper sediment is illustrated by elevated relative intensities of aliphatic compounds with low O/C ratios in the surficial WE-OM (orange to red color). Besides a change in the main organic matter source, differences in the reactivity of different organic matter types could also contribute to the molecular variations between WE-OM in the surface and deeper sediment. Saturated aliphatic compounds (group 5), which might contain fatty acids and carbohydrates, are considered as easily biodegradable components of marine organic matter and are quickly lost during early diagenesis (Freese et al., 2008). The higher biodegradability of saturated and unsaturated aliphatic compounds might contribute to their lower abundances in the deeper sediment compared to the sample from the surface sediments (Figure 3).

### Sources and bioavailability of organic matter in deeper sediments

The ^13^C depletion of TOC in sediments from the SMT area and below is consistent with an elevated proportion of terrestrial organic matter in the deeper sediments (Figure 1). TOC showed only minor variations in δ^13^C in deeper sediments, which is suggestive of similar sources, attributable to the high flux of terrestrial organic matter which was deposited during periods of heavy storms and disintegration of parts of the Helgoland Island (Hebbeln et al., 2003). Terrestrial organic matter consists of a high portion of complex O-rich structures e.g., lignin, tannin and cellulose. This is reflected in the higher abundance of O-rich aromatic and highly unsaturated compounds in the deeper sediment compared to the surface sediment (Figure 9A). Terrestrial organic matter is known to show greater recalcitrance in marine sediments compared to algal-derived organic matter (Andersen and Kristensen, 1992; Meyers and Ishiwatari, 1993; Meyers, 1994; Rontani et al., 2012). One reason for this could be pre-aging of terrestrial organic matter *en-route* the marine system or its higher susceptibility to encapsulation by accompanying minerals (Mayer, 1994; Keil, 2011; Lalonde et al., 2012; Riedel et al., 2013; Barber et al., 2014). In general, selective degradation strongly modifies the characteristics of residual organic matter in sediments (Meyers, 1994; Zonneveld et al., 2010). As microbes preferentially degrade the easily-utilizable portion of bulk organic matter, the more recalcitrant fractions selectively accumulate in deeper sediments (Cowie and Hedges, 1994; Wakeham et al., 1997). The generally higher abundances of CHO as well as CHO-N_1−2_ in the deeper sediments (Figure 2) suggest that a larger portion of the compounds represented by these formulae is relatively refractory. With respect to the molecular structures, highly unsaturated compounds (group 3) are likely to harbor a larger proportion of recalcitrant compounds as they are more abundant in deeper sediments (~58–64% in deeper sediments Figure 3). Changes in the abundance of different organic matter groups within the deeper sediments could be related to organic matter degradation. The percentage relative intensities of PCA and highly aromatic formulae (mostly CHO compounds) show a slightly decreasing trend below the SMT (Figure 3). This could be a result of a slow degradation of these formulae groups by microorganisms in the methanic zone. Similarly, the higher abundance of CHO-N_3−4_ formulae in the SMT and surface sediment relative to deeper sediments suggests that the N-rich compounds are preferentially degraded and therefore less abundant in the deeper sediments. This is consistent with reports of preferential degradation of N-rich organic matter in marine sediments (Cowie and Hedges, 1991; Freudenthal et al., 2001; Sinkko et al., 2013; Barber et al., 2014; Schmidt et al., 2014).

### Microbial populations and organic matter degradation in surface sediments

As surface sediments contained higher proportions of labile algal-derived aliphatic organic matter, bacterial groups belonging to *Gammaproteobacteria, Alphaproteobacteria*, and *Bacteroidetes*, often prominently detected during initial degradation of algal-derived organic matter in marine waters and sediments (Gutierrez et al., 2011; Teeling et al., 2012; Landa et al., 2014; Miyatake et al., 2014; Ruff et al., 2014) appeared to be more dominant therein. *Flavobacteriaceae*, the dominant members of the *Bacteroidetes* in surface sediments of our study site (Data Sheet S1, Tables 1, 2), have been consistently enriched in plankton-amended microcosm incubations as well as in natural phytoplankton blooms (Kirchman, 2002; Abell and Bowman, 2005; Bauer et al., 2006; Teeling et al., 2012). A recent study in an Arctic fjord (Smeerenburgfjord, Svalbard) has suggested a role in polysaccharide hydrolysis for members of the *Verrucomicrobia* phylum (Cardman et al., 2014). The occurrences of *Cyanobacteria, Acidobacteria*, and some members of the *Chloroflexi* (candidate class Ellin 6529; Data Sheet S1, Tables 1, 2) mainly in the surface sediments (Figure 7A) suggest that they may be better adapted to fresh organic matter. Dominant *Deltaproteobacteria* in surface sediments namely, *Desulfobulbaceae, Desulfuromonadaceae*, and *Desulfobacteraceae* (Data Sheet S1, Tables 1, 2), include various sulfate-, sulfur-, and metal-reducing bacteria that may specialize in the oxidation of low-molecular weight organic compounds fermentatively produced from upstream degradation of the heavier organic molecules (Lovley et al., 1993, 1995; Muyzer and Stams, 2008). Ammonia resulting from organic matter degradation is a potential substrate for the dominant *Thaumarchaeota* (mainly *Cenarchaeaceae*), which include known ammonia-oxidizing archaea such as *Nitrosopumilus maritimus* (Könneke et al., 2005) and *Candidatus* Nitrosopumilus koreensis (Park et al., 2010). Candidate division Parvarchaea also constitute a dominant archaeal group in surface sediment. However, no ecological role can be predicted for this candidate phylum due to lack of cultured members.

### Microbial populations and organic matter degradation in deeper sediments

The recalcitrant nature of organic matter in subsurface sediments may have selected for specific microbial populations capable of its utilization, resulting in lower bacterial and archaeal diversity compared to surface sediments (Figure 5). The diversities of Bacteria and Archaea in deeper sediments were mostly covered by the number of sequences analyzed in our study as respective rarefaction curves from deeper samples were already approaching plateau (Figures 6A,B). WE-OM from deeper sediments showed higher abundances of highly unsaturated compounds compared to the surface sediment (Figure 9A). These compounds may include CRAMs (Hertkorn et al., 2006) and some plant-derived materials rich in lignin/lignocellulosic molecules (Sleighter and Hatcher, 2008). Microbial populations dominant in deep sediments of our study site (*Chloroflexi*, candidate division JS1, MCG, and *Thermoplasmata*, Figures 7A,B), are consistent with those regularly found in marine subsurface sediments (Parkes et al., 2005; Biddle et al., 2006, 2008; Inagaki et al., 2006; Webster et al., 2007; Durbin and Teske, 2012; Schippers et al., 2012) and most of these microbial groups have been linked to heterotrophic metabolism (Biddle et al., 2006; Webster et al., 2007; Lloyd et al., 2013). In addition, the strong covariance of *Chloroflexi* (mainly *Dehalococcoidales*, ρ = 0.81 and candidate order GIF 9, ρ = 0.75), MCG archaea (mainly candidate order pGfrC26, ρ = 0.89), and *Thermoplasmata* (ρ = 0.81) to the depth profile of TOC in sediment core HE376-007-5 (Figure 8), suggests that these organisms are important for organic matter degradation in the deeper sediments of our study site as well. As organic matter source and input were relatively constant in the deeper sediments (> 30–530 cm, Figure 1), the observed shift of molecular signatures (mostly among CHO compounds) from high O/C and low to intermediate H/C ratios toward lower O/C and higher H/C ratios with increasing depth in the methanic zone (Figure 9B), are possibly a signature of selective organic matter degradation. Similar shifts have been observed in DOM degradation experiments (Kalbitz et al., 2003; Kim et al., 2006) and in subsurface sediments of peatlands where organic matter is considerably reactive (Tfaily et al., 2013, 2014, 2015), but not in marine subsurface sediments so far. A likely explanation is a microbial utilization of these O-rich highly unsaturated and aromatic compounds via potential reactions such as reduction or decarboxylation (Figure 9B). This offers an interesting new perspective to the range of organic matter potentially available for microbes in deep subseafloor as complex molecules such as for example, CRAM-like, lignin-like and tannin-like structures, as well as condensed aromatic molecules, have previously not been considered to be an important energy source for subsurface microbes. In line with our finding here, a role in fermentation of plant polymer building blocks (such as pyrogallol) has recently been predicted for a member of the candidate order GIF9 (Hug et al., 2013). In addition, members of the *Dehalococcoidia* are also known to be involved in the reductive degradation of substituted aromatic hydrocarbons (Alfreider et al., 2002; Fennell et al., 2004; Wasmund et al., 2014; Pöritz et al., 2015). Candidate order pGrfC26 are sub-grouped into the MCG-A or class 6 MCG (Meng et al., 2014) and are similar to Rice Cluster IV (Großkopf et al., 1998). These groups of MCG have been largely enriched in lignocellulose-amended cultures (Peacock et al., 2013) and may also have a role in the degradation of lignin monomers such as protocatechuate (Meng et al., 2014). Functional potential of organisms such as members of *Chloroflexi* and MCG in the degradation of aromatic compounds may have contributed to the molecular changes in CHO fractions of at least, PCA and aromatic formulae (group 1 and 2) in the deeper sediments of our study site. Potential for degradation of aromatic compounds were found in other *Chloroflexi*- and MCG- dominated subsurface sediments- e.g., in the Sonora Margin, Guayamas Basin, where genes responsible for degradation of aromatic hydrocarbons such as ethylbenzene and ethylphenol increased in proportion with depth (Vigneron et al., 2014).

The presence/higher abundances of candidate lineages such as OP1, OP8, WS3, and LCP-89 and *Planctomycetes* (mostly *Phycisphaerae*), *Elusimicrobia* (formerly Termite Group I), *Spirochaetes*, and *Actinobacteria* in deeper sediments in relation to the surface sediments suggest that they are better suited to the conditions or more important therein. *Firmicutes* in our site, mostly belonging to the *Bacillales* and *Clostridiales* (Tables 1–8 of Data Sheet S1), appear less selective as they are equally abundant in the surface and deeper sediments.

### Methanogenesis and AOM

Methanogenesis is the terminal step of organic matter degradation (Schink, 1997). The presence of methanogenic populations belonging to *Methanosarcinaceae* (harbor methylated C1 compounds, hydrogen and acetate utilizers), *Methanosaetaceae* (acetoclastic methanogenesis), *Methanomicrobiales* (hydrogenotrophic methanogenesis), and *Methanocellales* (hydrogenotrophic methanogenesis) suggest the potential for all three major pathways of methanogenesis in our site (Figure 7B). Methylotrophic methanogenesis has also been reported in members of the *Thermoplasmata* (Dridi et al., 2012; Paul et al., 2012; Iino et al., 2013; Poulsen et al., 2013). *Thermoplasmata* detected in this study all belong to the candidate order E2, a member (*Candidatus* Methanogranum caenicola) of which has recently been reported to reduce methanol to methane using hydrogen as an electron donor (Iino et al., 2013). Although in lower concentrations compared to surface sediments, methanol has been detected in pore waters of subsurface sediments of the Black Sea (Zhuang et al., 2014) and its source has been attributed to degradation of terrestrially-derived macromolecules such as lignin and pectin (Donnelly and Dagley, 1980; Schink and Zeikus, 1980). This may explain the strong covariance of *Thermoplasmata* with TOC (ρ = 0.812, *p* = 0.07) in deep sediment samples studied here. If the ability to utilize methylated C1 compounds is widespread among members of the candidate order E2, such methanogenic pathway may be very important in subsurface sediments as *Thermoplasmata* account for up to 17% of total archaeal populations in deeper sediments based on our sequencing method (Figures 7B, 8). However, analysis of *mcrA* genes and incubation studies on these sediment samples will be necessary to verify this hypothesis.

Potential for anaerobic oxidation of methane is reflected by the abundances of ANME populations (ANME-1 and ANME-2c). The highest combined abundance of ANME populations (~30% of archaeal populations) and the highest presence of *Deltaproteobacteria* (mostly *Desulfobacteraceae*) found in the SMT area are consistent with the distinctiveness of this zone as the active site for AOM coupled to sulfate reduction (Boetius et al., 2000). Nevertheless, potential for AOM in the Helgoland mud area may extend deeper into the methanic zone where iron reduction is occurring (Oni et al., 2015) suggesting the possibility of AOM coupled to iron reduction (Beal et al., 2009). ANME-1 were detected in all samples taken below 30–55 cm depth (4–8% of archaeal populations) in analogy to previous observations (Lloyd et al., 2011) and ANME-2c were also found in high proportion at 230-255 cm (16% of archaeal population).

## Conclusions

Our study suggests that the amount and composition of organic matter may influence the distribution of microbial populations in surface and deeper sediments of the Helgoland mud area (e.g., as seen in Figure 8). While nitrogen-rich, aliphatic organic compounds of presumed algal origin are mostly available for microorganisms in surface sediments, the subsurface sediments are dominated by aromatic and unsaturated phenolic compounds that presumably originate from terrestrial sources. Microorganisms dominating deeper sediments of our study site are consistent with those commonly found in other marine subsurface sediments. These dominant bacterial and archaeal populations are strongly correlated to the TOC content, suggesting involvement in degradation of organic matter in deeper sediments of our study site. Consistently, we observed molecular transformations in the water-extractable (potentially microbially-available) portion of bulk organic matter in subsurface sediments (particularly within the methanic zone) showing a shift from a higher abundance of O-rich molecules in the shallower subsurface (higher O/C ratio) toward a higher abundance of more reduced compounds (with higher H/C and lower O/C ratios). The assemblage of formulae corresponds to PCA, aromatics and highly unsaturated molecules that may include lignins, tannins, CRAM equivalents (groups 1–3), and is consistent with recent findings that O-rich compounds are also preferentially depleted in highly-reactive peatland subsurface sediments (Tfaily et al., 2015). We therefore conclude that organic matter with such oxygen-rich phenolic and aromatic compounds may be an important energy source for microorganisms inhabiting marine subsurface environments characterized by high depositional rates, such as the Helgoland mud area as well. The findings presented here thus shed more light on our understanding of molecular transformations for WE-OM in marine sediments and could accelerate ongoing efforts to culture microorganisms or enrich active microbial consortia in the marine subsurface sediments. In future, detailed analyses of functional genes linked to the degradation of algal polymers, aromatic and phenolic compounds in marine sediments would be necessary to confirm microbial involvement in observed depth-wise molecular transformations in organic matter composition.

### Conflict of interest statement

The authors declare that the research was conducted in the absence of any commercial or financial relationships that could be construed as a potential conflict of interest.

## Supplementary material

Figure S1 **ESI negative FT-ICR mass spectra of WE-OM extracted from the sediment cores of Helgoland mud area**. Largest peaks are contaminants (listed in the surfactant database: http://www.terrabase-inc.com//Surfactants.htm) and were removed from the final data set.

Figure S2 **FT-ICR mass spectra on the mass 385 Da for WE-OM with increasing sediment depth from top to bottom**. Symbols refer to different compound groups and homologous series. Homologous series are defined as the functional relationship between molecular formulae that differ by a specific mass difference equivalent to a chemical building block [in this case CH_4_ replaced by O (0.036 Da)].
