# Supplementary figures and images for: Microbial Communities and Organic Matter Composition in Surface and Subsurface Sediments of the Helgoland Mud Area, North Sea
Source: Front Microbiol. 2015 Nov 25;6:1290. doi: 10.3389/fmicb.2015.01290 (PMC4658423; doi:10.3389/fmicb.2015.01290)

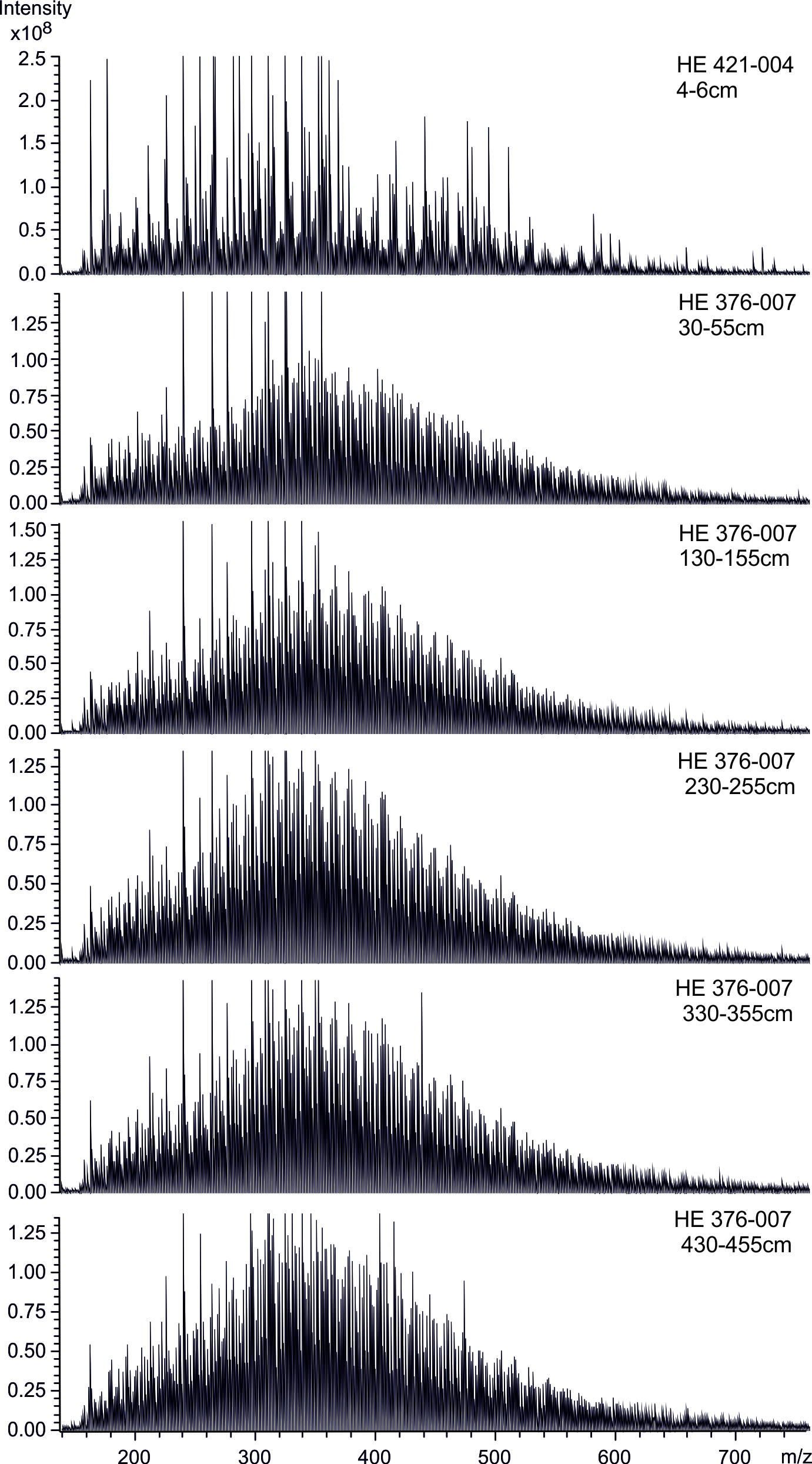

Supplement: Figure S1 — ESI negative FT-ICR mass spectra of WE-OM extracted from the sediment cores of Helgoland mud area. Largest peaks are contaminants (listed in the surfactant database: http://www.terrabase-inc.com//Surfactants.htm) and were removed from the final data set. [file Image1.JPEG]

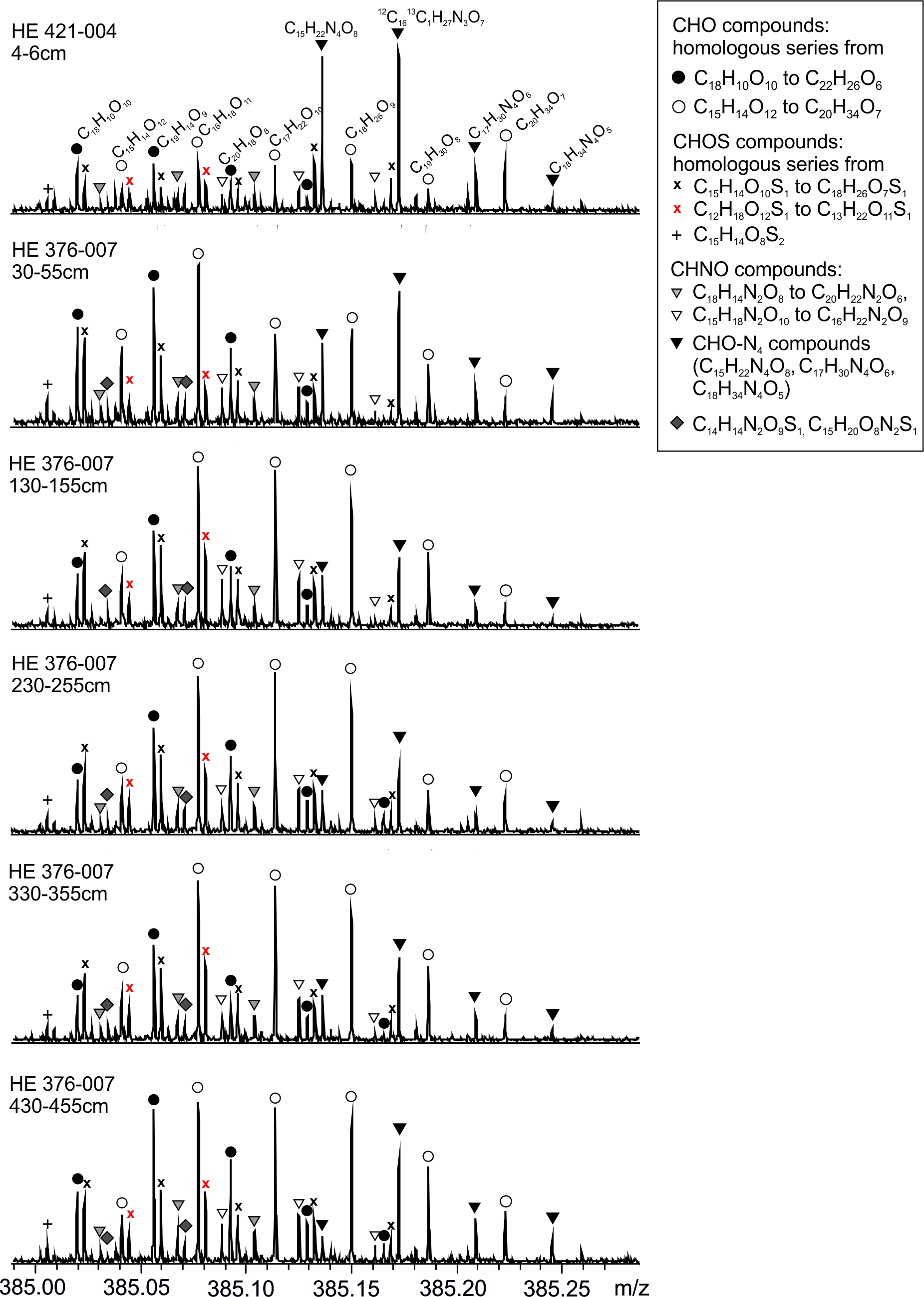

Supplement: Figure S2 — FT-ICR mass spectra on the mass 385 Da for WE-OM with increasing sediment depth from top to bottom. Symbols refer to different compound groups and homologous series. Homologous series are defined as the functional relationship between molecular formulae that differ by a specific mass difference equivalent to a chemical building block [in this case CH4 replaced by O (0.036 Da)]. [file Image2.JPEG]
